# Uncovering a pseudoscience: an analysis of ‘biological dentistry’ Instagram posts

**DOI:** 10.2340/aos.v83.40486

**Published:** 2024-04-24

**Authors:** Ana Maria Jucá, Olivia Santana Jorge, Yasmin Rosalin Moreira, Matheus Lotto, Tamires Sá Menezes, Thiago Cruvinel

**Affiliations:** a Department of Pediatric Dentistry, Orthodontics, and Public Health, Bauru School of Dentistry, University of São Paulo, Bauru, Brazil

**Keywords:** eHealth, misinformation, oral health, pseudoscience, social media

## Abstract

**Objective:**

This infodemiology study aimed to analyze characteristics of English-language Instagram posts on ‘Biological Dentistry’.

**Materials and Methods:**

Using CrowdTangle, we analyzed 500 ‘Biological Dentistry’ posts published on Instagram from May 2017 to May 2022. Two researchers assessed each post for facticity, motivation, author’s profile, sentiment, and interaction metrics. Statistical analysis was employed to compare interaction metrics between dichotomized categories of posts’ characteristics and determine predictors of misinformation and user engagement.

**Results:**

Over half of the posts (58.4%) were from health-related authors, and a considerable number contained misinformation (68.2%) or were financially motivated (52%). Sentiment was mostly negative or neutral (59.8%). Misinformation was associated with financial motivation (OR = 2.12) and health-related authors (OR = 5.56), while non-health-related authors’ posts associated with higher engagement (OR = 1.98). Reliable content, non-health-related authorship, and positive sentiment were associated with increased user interaction.

**Conclusion:**

Misinformation about ‘Biological Dentistry’ on Instagram is mainly spread by financially incentivized health-related authors. Yet, non-health-related authors’ posts resonate more with audiences, highlighting a nuanced relationship between content facticity, authorship, and engagement.

## Introduction

Internet users frequently turn to social media to seek advice and information on oral health issues [1, 2], despite concerns about the reliability and accuracy of such content [3]. There is a risk that individuals might encounter misinformation, leading to the adoption of behaviors detrimental to health, driven by unfounded beliefs [4, 5]. This phenomenon is illustrated by reduced uptake of influenza vaccines and increased consumption of non-fluoridated products, both of which are indicative of the spread of unreliable information [6, 7].

The proliferation of health misinformation on social media fosters an environment of uncertainty, facilitating the spread of pseudoscience – a body of knowledge or theory not grounded in scientific methodologies [8]. Key characteristics of pseudoscience include: (a) the widespread use of ad hoc hypotheses to justify negative research outcomes, (b) intentional evasion of the rigorous scrutiny provided by peer review, (c) a preference for confirmation over the critical process of falsification, (d) a marked detachment from the principles of fundamental or applied research, (e) an overreliance on anecdotal evidence, and (f) a reversal in the dynamics of proof, where proponents demand that critics disprove the efficacy of their methods [9].

Dentistry is not immune to the impact of misinformation and dubious practices [10, 11], such as ‘Biological Dentistry’, also referred to as ‘Holistic Dentistry’ or ‘Integrative Dentistry’ [12]. The International Academy of Biological Dentistry and Medicine (IABDM) defines ‘Biological Dentistry’ as an approach that promotes healing through team-based strategies, including mercury-safe dentistry, personalized testing for the biocompatibility of dental materials, and the diagnosis and treatment of dental and intra-oral conditions with an understanding of energy, electromagnetics, sound, light, acupuncture, homeopathy, nutrition, and appropriate detoxification methods for removing toxic heavy metals [13]. Quackwatch, a nonprofit organization dedicated to monitoring health fraud globally, categorizes Biological Dentistry as a dubious practice alongside other non-evidence-based methods such as chiropractic, chelation therapy, and craniosacral therapy [14].

Given the widespread use of social media for specific health information, it is essential to identify and assess posts related to pseudoscience for surveillance purposes [15]. This is vital for raising awareness among stakeholders about the potential risks to professional practice and for informing the development of policies aimed at curbing the dissemination of such content on social media platforms. Analyzing the vast amount of data generated by the creation and consumption of oral health information online can help to identify the needs of specific populations, guiding the planning and execution of health interventions [16–19]. Within this context emerged the concept of infodemiology, defined as ‘the science of distribution and determinants of information in an electronic medium with the ultimate aim to inform public health and public policy’ [20].

Therefore, this study aimed to scrutinize and delineate the characteristics of English-language Instagram posts related to ‘Biological Dentistry’, focusing on their facticity, motivation, author’s profile, and sentiment.

## Materials and methods

### Study design

**Figure 1:**
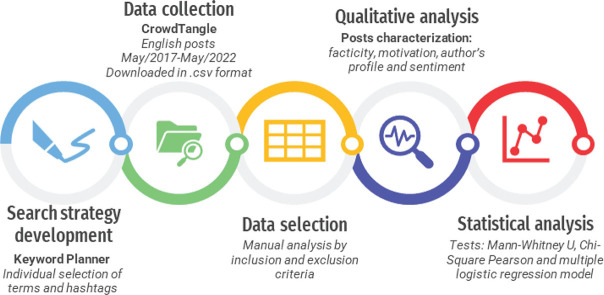
Summarized representation of study design.

This infodemiology study showcased 500 English-language Instagram posts about ‘Biological Dentistry’. Initially, 3,775 ‘Biological Dentistry’-related posts published between May 2017 and May 2022 were retrieved from CrowdTangle^TM^, sorted by users’ total interaction. Subsequently, two independent investigators (*OSJ and YRM*) qualitatively evaluated 500 of them using content analysis for the facticity, motivation, author’s profile, and sentiment. Finally, data were evaluated by descriptive analysis, Mann–Whitney U test, and multiple logistic regression models regarding interaction metrics. See the summarization of the study design in Figure 1.

### Ethics Declaration

The research did not require approval by the Ethics Committee on Human Research of the Bauru School of Dentistry because federal regulation does not apply to research that uses publicly available data on the internet. The raw data of this article were anonymized and publicized in the Figshare repository.

### Search strategy and data collection

CrowdTangle, a social media analytics tool provided by Meta Inc., allows publishers, journalists, researchers, and fact-checkers to track, analyze, and report on public content across Facebook, Instagram, Reddit, and X (formerly Twitter) [21, 22]. It offers metrics such as post counts, dates, profile information, post types, total interactions (sum of likes, comments, and views), and the overperforming score. Our group received exclusive access to CrowdTangle from Meta Inc. for researching health information disorders.

Considering the expected association between ‘Biological Dentistry’ and product marketing, Instagram was selected as the platform for this study due to its significant commercial orientation, distinguishing it from platforms such as Twitter, which prioritize communication over commerce.

The overperforming score ranks posts by their performance relative to their expected total interactions, taking into account the profile’s follower count and the performance of previous posts [23, 24]. To estimate expected interactions, the algorithm excludes the top and bottom 25% of the last 100 posts based on their performance, focusing on the middle 50% to calculate their expected total interactions over set intervals (e.g. 15 min, 60 min, 5 h). Scores above 1.0 indicate posts that performed well beyond the profile’s potential reach, while scores below -1.0 suggest underperformance.

The search strategy was developed from an exploratory analysis of keywords using the KeywordPlanner tool (Google Ads), responsible to generate a list of terms related to ‘biological dentistry’. The generated terms were tested individually and in combination in CrowdTangle to determine the search strategy that recovered the highest number of posts. The following search strategy was chosen: ‘biological dentistry’ OR #biologicaldentistry OR ‘holistic dentistry’ OR #holisticdentistry OR ‘functional dentistry’ OR #functionaldentistry OR ‘natural dentist’ OR #naturaldentist OR ‘natural dentistry’ OR #naturaldentistry OR ‘holistic dentist’ OR #holisticdentist OR ‘bio dentist’ OR #biodentist OR ‘biological dentist’ OR #biologicaldentist OR ‘organic dentist’ OR ‘integrative dentist’ OR #integrativedentist.

A .csv file was obtained from CrowdTangle, containing information about 3,775 Instagram posts. To refine the dataset, a filtering process was applied, focusing on English-language posts from the period spanning the last 5 years (May 2017 to May 2022). This time frame was selected based on the parameters established by Lotto et al. [11], aiming to analyze the most current data available while maintaining a robust sample size. To ensure the inclusion of Instagram posts with the greatest engagement, the posts were organized in descending order according to their total user interactions.

With the aim of selecting 500 posts, a comprehensive manual review of the collected links was individually undertaken. During this review, special attention was given to identifying instances of the term ‘biological dentistry’ or its equivalent (e.g. holistic dentistry, integrative dentistry), whether mentioned within the post’s text, hashtags or images, ensuring that the chosen posts were genuinely pertinent to ‘biological dentistry’, devoid of duplicates or content generated through reposting applications.

These posts were then anonymized by redacting names, profiles, and individuals’ eyes in images to maintain anonymity, systematically numbered, and organized in their original sequence in Google Slides (Google, Mountain View, CA, USA), later converted to a .pdf format. This methodical approach facilitated a consistent and ethical analysis of the content by various investigators at different times, eliminating discrepancies due to potential post modifications or deletions.

### Data analysis

#### Qualitative analysis

The qualitative analysis was conducted by two trained and calibrated independent investigators (*OSJ and YRM*). The training was conducted alongside an experienced researcher, involving the assessment and discussion of 10 posts based on available literature [11]. Subsequently, the two investigators were calibrated through the evaluation of 10% of the sample (*n* = 50) and the calculation of the Intraclass Correlation Coefficient (ICC) [25].

The process aimed to objectively and reliably ascertain the authors’ intentions, acknowledging that such determinations rely on subjective judgments based on the researchers’ perspectives [26]. The difficulty of distinguishing between unintentional errors and deliberate misinformation, as highlighted by Poe’s Law, underscores the challenges of relying solely on content cues to discern intent [27, 28]. Some scholars argue for the use of ‘disinformation’ specifically in cases where financial motives are evident, despite the classification challenges [29].

To avoid misinterpretation of authors’ intentions in spreading falsehoods, this study employs ‘misinformation’ as an umbrella term [30]. This designation serves as a comprehensive descriptor, preventing premature categorization and recognizing the difficulties in identifying the motivations behind the spread of deceptive information. Accordingly, posts were identified as misinformation if they propagated content that was demonstrably false or misleading, lacking a basis in scientific evidence [26, 31].

The investigators also assessed posts for underlying motivations and sentiment. The dissemination of misinformation online has been systematically categorized into social, psychological, financial, and political motives, according to the framework by Wardle and Derakhshan [26]. Social motivations relate to the desire to affiliate with certain groups; psychological motivations involve altering perceptions for prestige; financial motivations include profit from misleading content through advertising or sales; and political motivations aim to influence public opinion based on specific ideologies. This taxonomy elucidates the complex nature of misinformation spread and aids in understanding the varied motives behind it.

Prior studies have associated positive emotions with higher engagement rates on social media [32, 33]. Therefore, post sentiment was categorized as positive, neutral, or negative. Posts conveying positive emotions toward ‘Biological Dentistry’, such as expressions of joy or motivational content, were classified as positive. Descriptive posts about ‘Biological Dentistry’ without emotional language were deemed neutral. Posts expressing negative emotions, such as distress or fear related to ‘Biological Dentistry’, were classified as negative. The classification was based on the predominant sentiment as perceived by the investigators.

Following the qualitative analysis, any discrepancies in classification were resolved through discussion until a consensus was reached between the investigators. If consensus was unattainable, the final classification was made by a third senior researcher (*TC*).

### Statistical analysis

Statistical analysis was performed using the software SPSS version 28.0. Inter-examiner reliability was assessed by calculating the ICC, where values greater than 0.75 were considered indicative of good reliability for subsequent analyses.

Prior to statistical analysis, qualitative variables were dichotomized as follows: facticity (information or misinformation), motivation (financial or non-financial), author’s profile (health-related and non-health-related authors), and sentiment (positive or negative/neutral). Furthermore, continuous variables such as time of publication (measured in days), total interactions (count of interactions), and overperforming scores (measured as a score) were dichotomized based on their median values.

The normality of the data distribution was assessed using the Kolmogorov–Smirnov test, while homogeneity of variances was evaluated through the Levene test. Given the data’s non-normal distribution, comparisons of total interactions and overperforming scores across dichotomized categories were conducted using the Mann–Whitney U test. Moreover, multiple logistic regression models were utilized to investigate the relationships between facticity, total interactions, and overperforming scores with the variables mentioned above. Only variables that demonstrated significant Wald statistics in univariate regression models were included in the multivariate regression analyses. The absence of multicollinearity in the logistic regression models was verified by examining the correlation among independent variables (*r* < 0.7) and by calculating the Variance Inflation Factor (VIF < 2.0) [34]. A *P*-value of less than 0.05 was considered statistically significant for all analyses.

## Results

The ICC values ranged from 0.778 for sentiment to 0.937 for motivation, verifying the calibration of independent investigators for content analysis.

**Figure 2:**
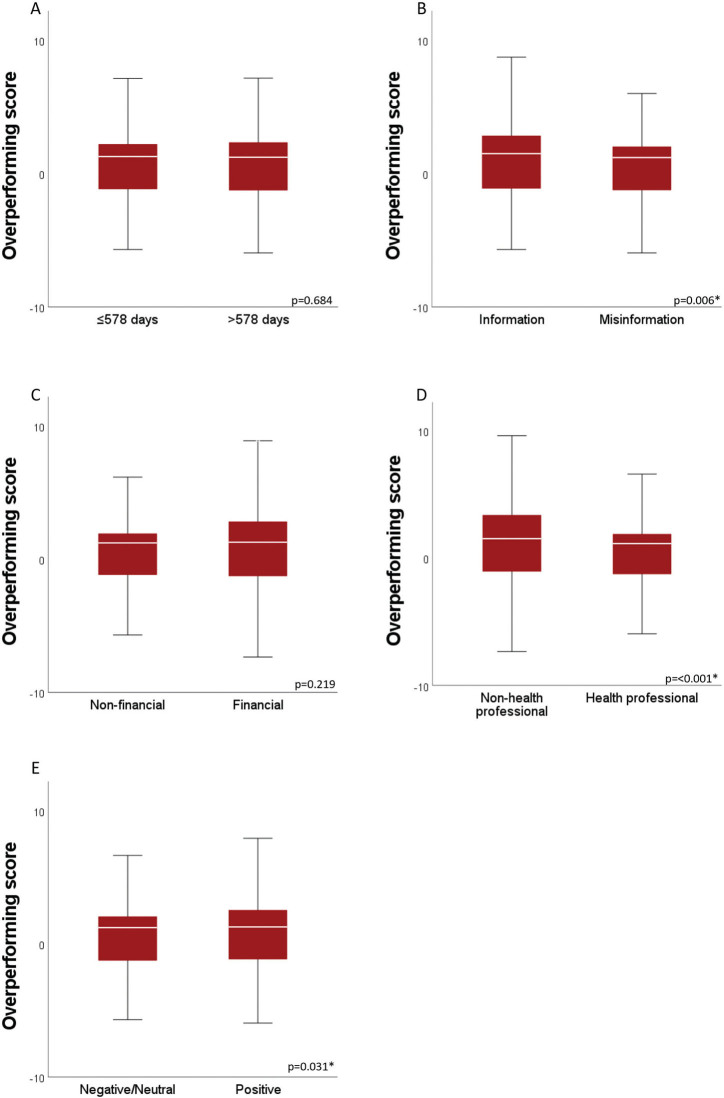
A comparison of medians (IQR) of overperforming scores for dichotomized categories of (A) time of publication, (B) facticity, (C) motivation, (D) author’s profile, and (E) sentiment (Mann–Whitney U test, *P* < 0.05).

**Figure 3:**
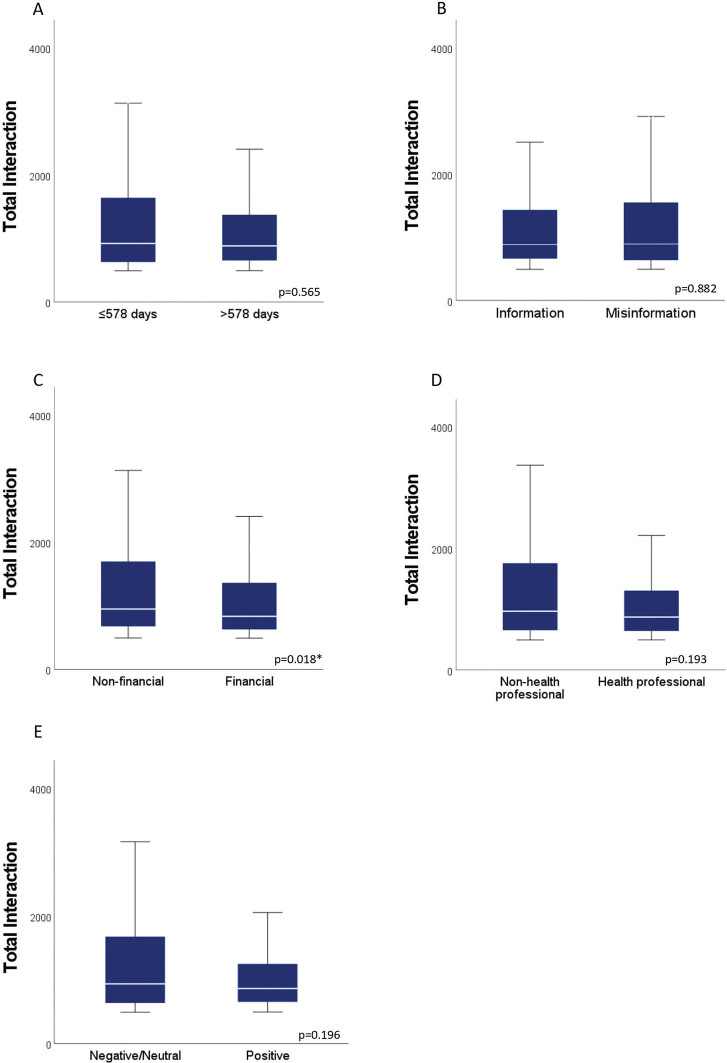
A comparison of medians (IQR) of total interaction for dichotomized categories of (A) time of publication, (B) facticity, (C) motivation, (D) author’s profile, and (E) sentiment (Mann–Whitney U test, *P* < 0.05).

Box 1 illustrates examples of the content found in the analyzed posts and their respective classifications. The findings revealed that a majority of the posts contained misinformation (68.2%) and were driven by financial motives (52%). Additionally, 58.4% of the posts were produced by health-related authors and 59.8% conveyed either a negative or neutral sentiment. It was observed that posts containing reliable information (*P* = 0.006), authored by individuals not related to health professions (*P* < 0.001), and expressing a positive sentiment (*P* = 0.031) were associated with higher overperforming scores (Figure 2). Posts motivated by non-financial reasons were linked to a greater number of total interactions (*P* = 0.017) (Figure 3).

Box 1. Examples of content related to different categories of facticity, motivation, and sentiment.

**Table:** 

Classification	Examples
**Facticity**	
Information	‘It’s time we stop treating the mouth and oral health as disconnected from the rest of the body… You simply cannot ignore dental health if you want good overall health’
Misinformation	‘In biological dentistry they view jaw cavitation as a major interference field, a common complication particularly for patients who are already immuno-suppressed with high levels of inflammation like chronic Lyme patients’ (sic)
**Motivation**	
Non-financial	‘Did you realise your teeth were so connected to your brain? It’s our nervous system & they make our body work’.
Financial	‘Say goodbye to Fluoride, Glycerin and Industrial chemicals!!! YESSS TruthPaste has been intentionally cultivated through years of research and artisan made with the highest grade, responsibly sourced organic essential oils…’ (sic)
**Sentiment**	
Positive	‘Stress free, anxiety free, pain free Dental Surgery. We treat everyone like family’
Negative	‘I’m hoping this message about teeth can resonate with someone out there that has Mercury Amalgam fillings. There fillings are poison and must be removed by a licensed holistic dentist and replaced with a natural filling’ (sic)
Neutral	‘How does holistic dentistry benefits your health? Click in my bio and read the full story’. (sic)

The analysis, as summarized in Table 1, indicates a significant correlation between health-related authorship and financial motivation with the dissemination of misinformation. These associations were further substantiated by multiple logistic regression models (Table 2), which demonstrated a significant positive relationship between financial motivation and misinformation (Odds Ratio [OR] = 2.12, 95% Confidence Interval [CI]: 1.40–3.21), as well as between health-related authorship and misinformation (OR = 5.56, 95% CI: 3.67–8.44). Furthermore, the models revealed a positive correlation between non-health-related authors and higher overperforming scores (OR = 1.98, 95% CI: 1.34–2.92). The analysis confirmed that multicollinearity did not influence the models, as indicated by the VIF and correlation outcomes: (1) for facticity [VIF(f1: author’s profile) = 1.000; VIF(f2: motivation) = 1.000; correlation(f1 × f2) = 0.123]; (2) for total interaction [VIF(f1: author’s profile) = 1.000; VIF(f2: motivation) = 1.023; VIF(f3: sentiment) = 1.023; correlation (f1 × f2) = 0.003; correlation (f1 × f3) = 0.014; correlation (f2 × f3) = −0.143]; (3) for overperforming score [VIF(f1: author’s profile) = 1.162; VIF(f2: facticity)v= 1.162; correlation(f1 × f2) = −0.357].

**Table 1:** A comparison of averages (± SD) and medians (IQR) of total interaction and overperforming score for dichotomized categories of time of publication, facticity, motivation, author’s profile, and sentiment (Mann–Whitney U test, *P* < 0.05).

	*n* (%)	Total Interaction			*n* (%)	Overperforming score		
		Average (± SD)	Median (IQR)	*P*		Average (± SD)	Median (IQR)	*P*
***Time of publication***							
≤ 578 days	250 (50.0%)	1,424 (± 1,393)	922 (1,010)	0.565	250 (50.0%)	1.07 (± 3.18)	1.29 (3.38)	0.684
> 578 days	250 (50.0%)	1,503 (± 4,200)	884 (714)		250 (50.0%)	2.50 (± 10.99)	1.24 (3.60)
***Facticity***							
Information	159 (31.8%)	1,293 (± 1,062)	884 (780)	0.882	159 (31.8%)	2.41 (± 9.14)	1.51 (3.99)	0.006*
Misinformation	341 (68.2%)	1,543 (± 3,715)	893 (931)		341 (68.2%)	1.49 (± 7.58)	1.21 (3.28)
***Motivation***							
Non-financial	240 (48.0%)	1,686 (± 4,358)	951 (1,023)	0.018*	240 48.0%)	1.30 (± 6.96)	1.24 (3.11)	0.219
Financial	260 (52.0%)	1,258 (± 1,105)	836 (736)		260 (52.0%)	2.24 (± 9.03)	1.30 (4.10)
***Author’s profile***							
Non-health professional	208 (41.6%)	1,767 (± 4,680)	965 (1,101)	0.193	208 (41.6%)	3.07 (± 9.96)	1.51 (4.44)	< 0.001*
Health professional	292 (58.4%)	1,247 (± 1,033)	869 (663)		292 (58.4%)	0.86 (± 6.34)	1.10 (3.15)
***Sentiment***							
Negative/Neutral	299 (59.8%)	1,379 (± 1,121)	937 (1,052)	0.196	299 (59.8%)	1.11 (± 6.49)	1.24 (3.35)	0.031*
Positive	201 (40.2%)	1,590 (± 4,742)	865 (595)		201 (40.2%)	2.78 (± 9.90)	1.29 (3.71)

**Table 2:** Multiple logistic regression models for facticity, total interaction and overperforming score.

	B^a^	S.E.^b^	Wald	P	OR^c^	95% IC	
						ICI	ICS
***Facticity (misinformation)***						
Author’s profile (health professional)	1.716	0.213	65.060	< 0.001*	5.562	3.666	8.439
Motivation (financial)	0.751	0.212	12.568	< 0.001*	2.120	1.399	3.212
Constant (y-intercept)	−0.486	0.180	7.320	0.007	0.615		
***Total interaction (> 893)***						
Author’s profile (health professional)	−0.326	0.183	3.169	0.075	0.722	0.504	1.033
Motivation (financial)	−0.283	0.182	2.406	0.121	0.754	0.527	1.077
Sentiment (positive)	−0.251	0.186	1.818	0.178	0.778	0.541	1.120
Constant (y-intercept)	0.422	0.180	5.502	0.019	1.525		
***Overperforming (> 1.27)***						
Author’s profile (non-health professional)	−0.684	0.198	11.892	0.001*	1.981	1.343	2.922
Facticity (misinformation)	−0.225	0.210	1.147	0.284	0.798	0.529	1.206
Constant (y-intercept)	0.538	0.175	9.428	0.002	1.713		

a Unstandardized coefficient.

b Standard error.

c Odds ratio.

## Discussion

This study offers preliminary insights into the accuracy, quality, and sentiment of social media posts on ‘Biological Dentistry’, identifying misinformation sources and impacts and suggesting strategies to mitigate its spread. While the bulk of Instagram posts were laden with misinformation and displayed neutral or negative sentiments, mainly from health-related authors, it was the posts conveying reliable content, stemming from non-health-related authors, and carrying positive sentiments that exhibited superior engagement performance. Moreover, posts driven by financial motivation or authored by health-related individuals were more frequently associated with misinformation. In contrast, those from non-health-related author profiles were more inclined to achieve higher overperforming scores.

The spread of health misinformation on the internet has been a persistent issue, with its impact notably intensified during the pandemic, especially across various social media platforms. This issue is particularly alarming in the context of ‘Biological Dentistry’, where misleading content is often disseminated by health-related profiles, thus undeservedly enhancing their credibility [35]. Despite the tendency of unreliable health-related information to attract more attention on social media [36–39], this study highlights a significant trend toward increased engagement with reliable information. This shift can be attributed to the study’s specific context, the professional community’s skepticism toward pseudoscience, and the limited accessibility of such information due to cost and unfamiliarity.

The absence of scientific evidence supporting alternative therapeutic approaches does not justify endorsing implausible or superstitious practices. Advocating for such practices distorts the dynamics of the burden of proof, a strategy commonly used by proponents of dubious claims [9]. Thus, it is crucial to maintain a strong scientific basis when evaluating and supporting therapeutic methods.

Posts on ‘Biological Dentistry’ predominantly expressed negative or neutral sentiments, reflecting concerns about the potential toxicity of fluoride and amalgam and the health risks associated with diverging from this practice’s principles. These sentiments were influenced by various factors, including the topic (e.g. vaccination, COVID-19, cancer, smoking) and the perspective taken (e.g. cure, medicine, treatment, disease, symptoms) [40–42]. The dental academic community has supported the reduction of amalgam use for environmental reasons and the development of alternatives to Bis-GMA-containing materials due to their potential estrogenic effects [43–45]. Yet, ‘biological dentists’ advocate against the use of amalgam and fluoride and oppose endodontic treatments, despite scientific evidence to the contrary [46–49].

Posts from non-health-related authors saw increased engagement, likely due to the emotional resonance of the content, which often reflects personal views and experiences, thus fostering user interaction [50–52]. Commercial posts also tend to attract more engagement due to their sales-driven language [53, 54], aligning with the goals of ‘Biological Dentistry’ in promoting specific treatments and products. Authors discussing ‘Biological Dentistry’ are frequently motivated by financial interests, seeking to persuade audiences of the purported natural and superior health benefits of their practices. This approach utilizes persuasive and emotionally charged rhetoric, which despite its imprecision, possesses considerable allure.

These findings can serve as inputs to train artificial intelligence systems in identifying false content associated with ‘Biological Dentistry’, subsequently implementing corrective measures to counter its propagation and holding accountable those responsible. This proactive approach could shield users from exposure to detrimental health practices, concurrently supporting evidence-based dental standards. Furthermore, investigations into health-related misinformation not only enrich our comprehension of the field but also furnish opportunities for interventions addressing various forms of digital illiteracy, empowering individuals and endowing them with autonomy over their digital information consumption.

This study faces some limitations that merit consideration. Firstly, the sample size was confined to 500 posts, constrained by the complexities inherent in manual content analysis. This limitation reflects the methodological challenges of labeling datasets by hand, a common issue in previous research endeavors [11, 55]. Although it was impractical to assess the entire volume of posts retrieved by CrowdTangle, our analysis was selectively focused on posts that demonstrated genuine user engagement, identified from a comprehensive list ranked by interaction levels. Secondly, our classification of content as either false or misleading did not differentiate between misinformation and disinformation, primarily due to the difficulty in discerning the intentions of the original authors [26]. This delineation is critical, as misinformation unintentionally misleads, whereas disinformation is deliberately deceptive. Thirdly, the analysis was limited to English-language posts, a restriction that may not fully account for cultural variances that could influence the content’s interpretation and impact. Since English is the predominant global language, it serves as a primary conduit for the dissemination of health misinformation among users. Fourthly, the inherent subjectivity in analyzing content, especially regarding the classification of motivation and sentiment, presents another challenge. To counteract this, the classification process was not solely reliant on literature but was augmented by duplicate assessments and consensus among trained and calibrated investigators, thereby enhancing the reliability of our findings [26, 56]. Fifthly, this study concentrated exclusively on quantitative metrics of post interactions and did not delve into the qualitative examination of user comments, which could provide deeper insights into the audience’s reaction to misinformation. Such qualitative analysis requires additional methodological approaches, highlighting the need for future research to explore the effects of misinformation about ‘Biological Dentistry’ on social media engagement more comprehensively. Lastly, the potential influence of automated bots on interaction data cannot be ignored. While it is plausible that some authors might have employed specialized services to artificially boost their interaction metrics, the extent to which bots have influenced the data analyzed in this study remains uncertain. Previous studies have identified a significant use of bots for political purposes, a finding that starkly contrasts with the motivations uncovered in our investigation [57, 58]. This discrepancy underscores the complexity of assessing bot impact within the specific context of ‘Biological Dentistry’ misinformation.

## Conclusion

In summary, the substantial presence of misinformation, the widespread financial motivation behind posts, and the volume of misleading content authored by health-related professionals highlight the critical need for enhanced scrutiny in verifying the reliability of online health information. This is particularly acute in digital environments where commercial motives frequently shape content. These analyses reveal that financial motivations and the involvement of health-related authors are significant predictors of misinformation dissemination. Conversely, posts from non-health-related authors are associated with increased user engagement with ‘Biological Dentistry’ content on Instagram. These observations underscore the imperative for more rigorous oversight and the development of strategies to mitigate the impact of financially driven and misleading health information in online communities.

## Declarations

### Data availability statement

The datasets generated and/or analyzed during this study are available in the Figshare repository, available in https://figshare.com/s/6714c9696a34d8fe9e73.
